# Social relationships and their association with the functional capacity of older Chilean adults: longitudinal evidence

**DOI:** 10.1186/s12877-024-05184-x

**Published:** 2024-07-19

**Authors:** Letícia de Albuquerque Araújo, Gloria Icaza, Carlos Márquez, Cecilia Albala

**Affiliations:** 1 School of Nutrition, Faculty of Health Sciences, Catholic University of Maule, Carmen 684, Curicó, Chile; 2 School of Public Health, Faculty of Medicine, University of Chile, Av. Independencia 939, Santiago, Chile; 3 Institute of Mathematics and Physics, University of Talca, Av. Lircay, S/N, Talca, Chile; 4 Aging Core, Public Nutrition Unit, Institute of Nutrition and Food Technology (INTA), University of Chile, Av. El Líbano 5524, Macul, Santiago, Chile

**Keywords:** Healthy aging, Relationships, Social participation, Social network, Older adults

## Abstract

**Background:**

Functional capacity is recognized as a central factor for health in old age and not all studies that seek to clarify the role of social relationships in functional capacity are conclusive. The subject has only been studied in a limited way in Latin America, a region that is aging prematurely, with evidence primarily from developed countries, which have experienced a more gradual aging of their population. This longitudinal study aimed to determine how aspects of social relationships impact the functionality of older Chileans.

**Methodology:**

We conducted a cohort study of 2,265 people aged 60 years or older who lived in the community and resided in Greater Santiago, Chile. Five aspects of social relationships were considered at baseline (participation in groups, clubs, or organizations; number of people in the household; participation in recreational activities; perception of material support, help or advice, and marital status), from which a cluster analysis by conglomerate was performed and used as the exposure of interest. Functional limitation (FL) was the dependent variable, classified as a limitation in at least 1 basic activity of daily living or 1 instrumental activity or 2 advanced activities. The control variables considered were: sex, age, educational level, multimorbidity, depression and years of follow-up. Survival analyses using a Cox proportional hazard regression and multilevel logistic regressions (person level and follow-up wave level) were performed.

**Results:**

The identified clusters were four: “without social participation and does not live alone”; “without a partner and without social participation”; “no perception of support and no social participation”; “with participation, partner and perception of support”. Social relationship clusters predicted FL incidence and FL reporting during follow-up. Being in the clusters "without social participation and does not live alone" and "without partner and without social participation" were risk factors for incident FL and report of FL during follow-up, compared to being in the reference cluster "with participation, partner and perception of support.

**Conclusions:**

In summary, our study showed that participating in social organizations, not living alone and having a partner are protective factors for presenting and developing functional limitation in old age for community-living Chileans in an urban area.

**Supplementary Information:**

The online version contains supplementary material available at 10.1186/s12877-024-05184-x.

## Introduction

Old age is a process that is lasting longer and longer and longevity has given dynamism to the experience of aging, so that it is not just an unalterable stage [1]. Under the life course approach, it is understood that aging is a process that occurs from birth and that in old age the impact of the different determining factors of health accumulates [2]. Within the framework of healthy aging [3], functional capacity is recognized as a central factor for health in old age, so that a healthy old age is not understood as the absence of disease, but rather greater independence to do what is significant for the person, even in the presence of multimorbidity.

Among external factors that form the context of a person's life are social relationships and are one component of healthy aging [3]. Social relationships can be understood as links between people, which are influenced by individual and collective factors. Relationships can impact health based on behavioral, psychosocial and physiological mechanisms [4–7]. For example, in a previous study, we determined that social integration was an important determinant of fruit and vegetable consumption in old age [8]. Being the maintenance of functionality a central physical aspect for the independence of an older person, its study has focused on biological aspects and risk factors.

Not all studies that seek to clarify the role of social relationships in functional capacity are conclusive. Some have found that better indicators of social relationships predict better functional capacity [9–15], while other studies could not confirm such a finding [16, 17]. The subject has only been studied in a limited way in Latin America, a region that is aging prematurely [18], with evidence primarily from developed countries, which have experienced a more gradual aging of their population. The evidence in Latin America is predominantly from cross-sectional studies or longitudinal studies that have evaluated only one aspect of social relations as an exposure [19–22].

Chile is a country that has experienced rapid aging of its population since the 1950s, which has been attributed to socioeconomic and health changes [23]. Today, Chile is the country with the highest life expectancy at birth (81.2 years in 2023) within Latin America [24]. In this context, this study seeks to answer the question how social relationships impact the functionality of older Chileans, based on data from a long follow-up.

## Methods

We conducted our study within the ALEXANDROS (Active Life Expectancy, Aging and Disability Related to Obesity Study) study of older people living in the community in Santiago, Chile, previously described [25]. Briefly, Alexandros is a longitudinal study of people ≥ 60y including a first group of 1302 people born before 1940 recruited in the frame of the Santiago SABE study [26] and a second group of 963 people born between 1940–1948 recruited between 2005 and 2008, randomly selected from 28 Public Primary healthcare center registries.

Thus, this study included 2265 Alexandros study participants followed between 2000 and 2016 in relation to functional status, with one, two or three follow-ups. 1,803 older people were evaluated on more than one wave, of which 449 of these died before their second wave. The cohorts are dynamic and have grown over time. Exclusion criteria were not considered [27]. Participants were interviewed by professionals trained for questionnaire standardization and provided informed consent.

### Exposure variable

Considering five aspects of social relations in the baseline, a hierarchical cluster analysis was carried out. We aimed to integrate several aspects of social relations and to study how older people would be grouped according to these characteristics. We considered the following five aspects: housing arrangement (number of people in the household, from 1 to 13); marital status (married or in a relationship; single; widowed; separated or annulled); participation in community group, club or organization (no/yes); participation in recreational activities with other people (playing cards, dominoes, knitting, gardening, listening to music, reading, going to the movies; no/yes); perception of availability of material help, company or advice (no/yes). We created dichotomous variables for each category of the original variables, used Ward's analysis for the agglomeration method and simple matching as the measure of similarity for dichotomous data. The dendrogram describing the first twenty formed groups was observed (See Fig. 1 of Supplementary Data) and the point at which four different groups branched off was chosen.

### Outcome variable

Functional limitation was measured through the performance reported in six basic activities of daily living (ADL), seven instrumental activities of daily living (IADL) and five advanced activities of daily living (AADL) (Table 1). ADLs are based on Katz et al**.** [28], the IADLs at Lawton & Brody [29] and the AAVDs at Nagi & Marsh [30] and Rosow & Breslau [31]. According to criteria established by Albala et al. [32], older adults with limitation in at least one ADL or one IADL or two AADL were be considered with functional limitation.

**Table 1:** Activities considered for functional capacity classification

Basic activities of daily living	Instrumental activities of daily living	Advanced activities of daily living
Take a bath	Prepare hot food	Walk several streets
Dressing	Manage your own money	Climb a flight of stairs
Bathroom use	Leave home alone	Pick up a coin from a table
Transfer	Shopping	Bending down to pick up an object
Walk on a level surface	Make or receive phone calls	Lifting or carrying an object weighing more than 5 kilos
Feeding	Do light housework	
	Organize and take your medications	

The control variables considered were sex (male/female), age (in years), educational level (no formal studies/1 to 8 years/9 to 12 years/more than 12 years), multimorbidity (two or more chronic diseases reported: high blood pressure, diabetes, reported heart attack, lung disease, stroke, cancer, osteoporosis, rheumatoid diseases; reference category: no multimorbidity), risk of depression (> 5 points on the Geriatric Depression Scale; reference category: ≤ 5 points) [33] and years of follow-up (from baseline and last follow-up).

### Statistical analysis

Sociodemographic and health variables of older persons were described by sex using Fisher's exact test for categorical variables and the Kruskal–Wallis test for continuous variables. To analyze the incidence of functional limitation in people without functional limitation at baseline, Kaplan–Meier survival curves were constructed, considering time until the appearance of functional limitation between baseline and last follow-up as the censoring variable. Cox regression models were used to study the incidence of functional limitation by cluster in a multivariate analysis. To study functionality in the waves of follow-up and its relationship with clusters, considering the subject and evaluation wave (from 1 to 4), multilevel logistic regression models were estimated using functional limitation (no/yes) as the dependent variable. All analyses considered a significance level of 0.05 and were carried out in Stata version 14.

## Results

Four clusters were identified based on social relationships (Table 2):

**Table 2:** Characterization of the clusters according to aspects of social relations

Aspect of social relationships	Cluster 1	Cluster 2	Cluster 3	Cluster 4	
	Without social participation and does not live alone (*n* = 584)	Without a partner and without social participation (*n* = 734)	No perception of support and no social participation (*n* = 428)	With participation, partner and perception of support (*n* = 497)	*p*-value^2^
**Participates in recreational activities**^**1**^					
** No**	46 (11.4%)	327 (53.5%)	134 (39.0%)	5 (1.6%)	< 0.001
** Yes**	356 (88.6%)	283 (46.5%)	208 (61.0%)	304 (98.4%)	
**Participates in groups, clubs or organizations**					
** No**	584 (100.0%)	678 (92.4%)	422 (99.1%)	0 (0.0%)	< 0.001
** Yes**	0 (0.0%)	56 (7.6%)	4 (0.9%)	497 (100.0%)	
**Marital Status**					
** Married or in couple**	563 (96.2%)	139 (19.3%)	189 (44.4%)	311 (62.3%)	< 0.001
** Single**	0 (0.0%)	87 (12.0%)	47 (11.0%)	38 (7.6%)	
** Divorced**	7 (1.2%)	162 (22.4%)	49 (11.4%)	43 (8.6%)	
** Widowed or has lost a partner**	14 (2.6%)	333 (46.3%)	141 (33.2%)	105 (21.4%)	
** Number of people in household (SD)**^**4**^	4.3 (2.1)	3.5 (2.0)	3.5 (2.2)	3.7 (1.9)	< 0.001^3^
**Perception of support: material, company or advice**					
** No**	1 (0.2%)	1 (0.1%)	154 (98.7%)	47 (10.2%)	< 0.001
** Yes**	582 (99.8%)	725 (99.9%)	2 (1.3%)	413 (89.8%)	
Lives alone*					
** Yes**	0 (0.0%)	104 (14.2%)	84 (19.5%)	39 (7.8%)	< 0.001
** No**	584 (100.0%)	630 (85.8%)	344 (80.5%)	458 (92.2%)	

^*^Variable not included in cluster formation, provided for descriptive purposes only

^1^Playing cards, dominoes, knitting, gardening, listening to music, reading, going to the movies

^2^Fisher's exact test for categorical variables and Kruskal–Wallis test for continuous variable

^3^Number of people in the household differs only between the first cluster and the others

^4^SD: standard deviation

- Cluster 1, “Without social participation and does not live alone” (*n* = 584): Persons who mostly participate in recreational activities, but not in groups or organizations. It is the cluster with the highest proportion of people who are married or who live with a partner (not alone), have support and has the highest average number of people in the household.
- Cluster 2, “Without a partner and without social participation” (*n* = 734): Persons who mostly do not participate in groups or organizations, do not have a partner widowed, single or divorced), may or may not participate in recreational activities, and receive support. Although most do not live alone, this cluster has a higher proportion of individuals who do.
- Cluster 3, “No perception of support and no social participation” (*n* = 428): This group does not participate in groups or organizations and does not perceive social support. Although many are married or living with a partner, it has the highest proportion of individuals living alone.
- Cluster 4, “With participation, partner and perception of support” (*n* = 497): This group participates in both recreational activities and groups or organizations. Most are married or live with a partner, receive social support, and do not live alone.

The clusters were named according to their characteristics in relation to aspects of social relations and, given these characteristics, Cluster 4, "With Participation, Partner, and Perception of Support," was used as the reference group for further analyses.

Of the 2,265 subjects in the study, 67.3% were women, and 68.4% were aged 60 to 69 years at baseline, with 58.6% having 1 to 8 years of education. Regarding social relationships, 69.3% reported participating in recreational activities, while 24.8% participated in groups, clubs, or organizations. Most participants did not live alone (90%), and 89.6% received material help, company, or advice. Regarding marital status, 53.8% were married at baseline, while 26.8% were widowed or had lost a partner (Table 3).

**Table 3:** Sociodemographic and health characteristics of the sample at baseline by sex

Characteristic	Male (*n* = 733)	Female (*n* = 1.531)	Total	p*
**Average age, Years (SD)**	68.6 (6.5)	69.3 (7.2)	69.1 (7.0)	0.0281
**Educational level**				
No formal studies	88 (12.4%)	155 (10.4%)	243 (11.1%)	0.04
1 to 8 years	399 (56.2%)	888 (59.7%)	1.287 (58.6%)	
9 to 12 years	140 (19.7%)	318 (21.4%)	458 (20.8%)	
> 12 years	83 (11.7%)	127 (8.5%)	210 (9.6%)	
** Participation in recreational activities**^**1**^	389 (68.9%)	783 (69.5%)	1.172 (69.3%)	0.792
** Participation in groups, clubs or organizations**	145 (19.7%)	419 (27.3%)	564 (24.8%)	< 0.001
**Marital Status**				
** Married or in couple**	477 (65.8%)	726 (48.0%)	1.203 (53.8%)	< 0.001
** Single**	34 (4.7%)	138 (9.1%)	172 (7.7%)	
** Divorced**	72 (9.9%)	190 (12.6%)	262 (11.7%)	
** Widowed or has lost a partner**	142 (19.6%)	458 (30.3%)	600 (26.8)	
Live alone*	667 (90.6%)	1.379 (89.7%)	2.046 (90.0%)	0.474
** Number of people in household (SD)**^**2**^	3.8 (0.08)	3.7 (0.05)	3.8 (0.05)	0.3064
** Perception of support (material, company or advice)**	568 (88.6%)	1.177 (90.0%)	1.745 (89.6%)	0.328
** Depression**	141 (19.9%)	424 (28.9%)	565 (26.0%)	< 0.001
Multimorbidity**	323 (44.3%)	852 (55.8%)	1.175 (52.0%)	< 0.001
**Functionality in Wave 1**				
Limitation in at least 1 ADL	115 (15.6%)	256 (16.6%)	371 (16.3%)	0.538
Limitation in at least 1 IADL	241 (32.7%)	404 (26.3%)	645 (28.4%)	0.001
Limitation in at least 2 AADLs	276 (37.5%)	708 (46.0%)	984 (43.2%)	< 0.000
Functional limitation (general criterion)	398 (54.1%)	847 (55.1%)	1.245 (54.8%)	0.655

^*^Fisher's exact test for categorical variables and Kruskal–Wallis test for continuous variable

^**^Two or more reported chronic diseases

^1^playing cards, dominoes, knitting, gardening, listening to music, reading, going to the movies

^2^*SD* standard deviation

In terms of health and lifestyle characteristics, 26% of the sample had depression at baseline, and 52% reported multimorbidity, both conditions being more prevalent among women. At baseline, 55% had functional limitation, with no significant gender difference. In ADLs, 13.3% presented a limitation in at least one (with no differences by sex), while 28.4% presented limitation in at least one IADL, with prevalence being higher in men. Finally, in AADLs, 43.2% of the older adults presented limitations in two or more types of advanced activities, being more prevalent among women, as described in detail in Table 4.

**Table 4:** Sociodemographic and health characteristics of the sample at baseline by cluster of social relations

	Cluster				
	Without social participation and does not live alone	Without a partner and without social participation	No perception of support and no social participation	With participation, partner and perception of support	*p*^1^
**Female**	337 (57.6%)	528 (71.4%)	289 (67.4%)	370 (74.1%)	< 0.001
**Average age**					
** Years (SD)**	67.9 (6.1)	70.3 (7.8)	70.4 (8.0)	67.2 (4.7)	< 0.001^2^
**Educational level**					
No formal studies	30 (5.4%)	120 (16.8%)	63 (15.0%)	28 (5.8%)	< 0.001
1 to 8 years	325 (58.1%)	426 (59.6%)	251 (59.9%)	270 (55.6%)	
9 to 12 years	134 (24.0%)	121 (17.0%)	79 (18.9%)	121 (24.9%)	
> 12 years	70 (12.5%)	47 (6.6%)	26 (6.2%)	67 (13.8%)	
**Depression**	100 (17.2%)	204 (29.1%)	163 (39.4%)	98 (20.4%)	< 0.001
**Multimorbidity**	277 (47.6%)	387 (52.8%)	228 (53.4%)	276 (55.5%)	0.057
**Functionality in Wave 1**					
Limitation in at least 1 BADL	55 (9,4%)	167 (22,8%)	84 (19,6%)	46 (9,3%)	< 0.001
Limitation in at least 1 IADL	124 (21.2%)	285 (38.8%)	150 (35,1)	79 (15.9%)	< 0.001
Limitation in at least 2 AADLs	196 (33.6%)	377 (51.4%)	221 (51.6%)	178 (35,8%)	< 0.001
Functional limitation (general criterion)	272 (46.6%)	468 (63.8%)	267 (62.4%)	210 (42.3%)	< 0.001

^1^Fisher's exact test for categorical variables and Kruskal–Wallis test for continuous variable

^2^Age differs between all clusters

**Fig. 1:**
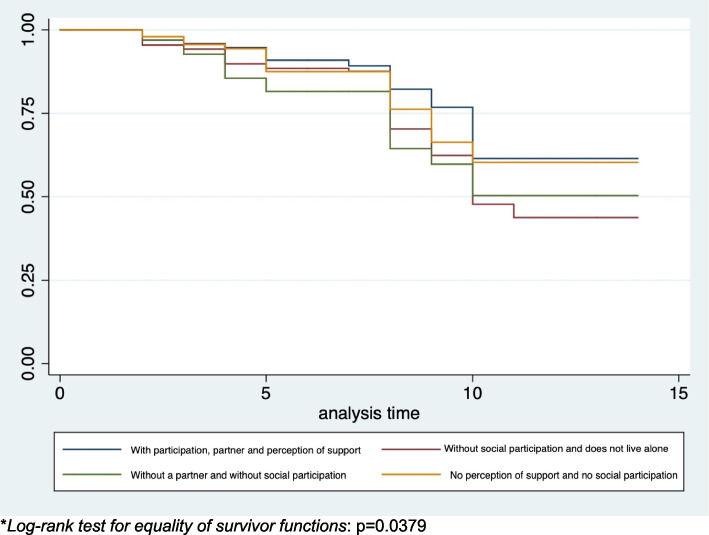
Kaplan–Meier curves for incidence of functional limitation between baseline and last follow-up

The total follow-up time of the sample in relation to functional status was 4254 years, from 2 to 14 years, with a median of 6 years and an interquartile range of 4 to 9 years. Of the 687 older people without functional limitation at baseline and with at least one subsequent evaluation, 140 (20.4%) presented functional limitation at their last follow-up, thus the incidence density of functional limitation was 0.033 person-year (95%CI 0.028–0.039). The Kaplan Meier curve for the incidence of functional limitation shows that the clusters "without social participation and do not live alone" and "without a partner and without social participation" had a higher incidence of FL, compared to the reference cluster “with participation, partner and perception of support “, at the end of follow-up (Fig. 1).

The survival analysis for the incidence of functional limitation adjusted for the control variables showed (Table 5) that being in the clusters "without social participation and does not live alone" and "without a partner and without social participation" compared to being in the cluster "with participation, partner and perception of support” is a risk factor for developing functional limitation between baseline and last follow-up.

**Table 5:** Cox regressions for incidence of functional limitation between baseline and last follow-up

Incidence of functional limitation	Crude model HR (95% CI)	Model with age and sex HR (95% CI)	Model with sociodemographic and health variables HR (95% CI)
**N**	686	686	676
**Clúster**			
With participation, partner and perception of support (reference)	1	1	1
Without social participation and does not live alone	1.63 (1.03–2.60)**	1.52 (0.96–2.23)	1.59 (0.99–2.54)
Without a partner and without social participation	1.86 (1.17–2.95)*	1.74 (1.10–2.78)**	1.80 (1.12–2.91)**
No perception of support and no social participation	1.29 (0.72–2.32)	1.29 (0.72–2.32)	1.37 (0.76–2.47)
**Age, years**		1.08 (1.05–1.11)*	1.08 (1.05–1.12)*
**Female sex**		1.14 (0.78–1.69)	1.16 (0.79–1.72)
**Educational level**			
No formal studies			1
1 to 8 years			1.38 (0.65–2.92)
9 to 12 years			0.87 (0.38–2.02)
> 12 years			1.44 (0.59–3.55)
**Multimorbidity**			1.13 (0.80–1.43)
**Depression**			0.79 (0.43–1.43)

^*^p < 0.001

^**^p < 0.05; HR: hazard ratio; CI: confidence interval

The multilevel analysis for functional status (Table 6) shows that, when adjusting for control variables, compared to older people in the reference cluster (with participation, partner and perception of support) being in the clusters "without participation and does not live alone” and “without a partner and social participation” are risk factors for functional limitation during follow-up. Finally, interactions between all adjustment variables and the clusters were studied. We did not observe any significant moderating factors.

**Table 6:** Multilevel logistic regression analysis for functional limitation variable

Functional limitation (yes)	Crude model OR (95% CI)	Model with sociodemographic variables OR (95% CI)	Model with sociodemographic and health variables OR (95% CI)
**N**	**4.567**	**4.481**	**3.562**
**Cluster**			
With participation, partner and perception of support	1	1	1
Without social participation and does not live alone	1.43 (1.11–1.85)*	1.35 (1.06–1.72)**	1.43 (1.10–1.85)**
Without a partner and without social participation	3.22 (2.50–4.15)*	2.06 (1.62–2.62)*	1.75 (1.35–2.26)*
No perception of support and no social participation	2.73 (2.05–3.64)*	1.83 (1.40–2.40)*	1.33 (0.98–1.80)
**Age (years)**		1.10 (1.08–1.11)*	1.10 (1.08–1.13)*
**Female sex**		1.13 (0.94–1.36)	1.12 (0.91–1.38)
**Educational level**			
No formal studies		1	1
1 to 8 years		0.39 (0.29–0.53)*	0.44 (0.30–0.64)*
9 to 12 years		0.20 (0.14–0.29)*	0.27 (0.18–0.41)*
> 12 years		0.21 (0.14–0.31)*	0.28 (0.17–0.45)*
**Multimorbidity**			1.59 (1.31–1.93)*
**Depression**			2.04 (1.63–2.56)*
Years of follow-up			0.96 (0.94–0.99)**
Constant (SD)	0.40 (0.04)*	0.003 (0.001)*	0.0009 (0.0008)*
**Random effects**			
Variance between subjects (SD)	1.71 (0.22)	1.18(0.18)	0.91 (0.16)
Residual intraclass correlation (SD)	0.34 (0.03)	0.26 (0.03)	0.22 (0.03)

*OR* odds ratio, *SD* standard deviation

^*^p < 0.001

^**^p < 0.05

## Discussion

The present longitudinal study among older community-living Chileans identified four clusters of social relationships, which differed mainly in social participation, marital status and perception of support. We found that those in the cluster "with participation, partner and perception of support" had a lower risk of presenting functional limitation in old age compared to older people in the clusters "without social participation and does not live alone” and “without a partner and without social participation”. People in the cluster “without a partner and without social participation” also had a higher risk of developing functional limitation during the follow-up compared with the reference cluster.

There are many differences between the clusters used in our study with respect to the aspects of social relations. Participating in groups, clubs or organizations and having a partner are two important characteristics must be taken into account when interpreting the results and it is an advantage of using the cluster or pattern approach [34] as many times these complexities cannot be seen in research focused on variables (for example, marital status alone).

The literature in Chile on this subject is limited. However, a longitudinal study with Chilean older adults found no differences in life expectancy without functional limitations in ADL (Activities of Daily Living) or in life expectancy with functional limitations in ADL between those who participated in social organizations and those who did not, which contrasts with the findings of the present study [35]. It is important to emphasize that the criteria for classifying functional limitation used in our study encompasses ADL, IADL, and AAVD, and this difference in results may reflect this difference. In addition, our exposure variable is a cluster and not the individual variable of social participation. Studies in Japan with older people living in the community have found that social or civic participation is a protective factor for decreased functional capacity and disability, although not all found this relationship [16, 36–38]. A systematic review of longitudinal studies by Stuck et al. [39] similarly found that low or no participation in social activities is a risk factor for functional limitation. In Mexico, a cross-sectional study analyzed clusters of social relationships in the older adult population and found that only the cluster of widows without social participation was a risk factor for functional limitation [20].

Regarding the greater risk for the occurrence and incidence of functional limitation in the subjects of the cluster "without a partner and without social participation", the results of previous studies on the effect of marital status on functionality are inconclusive. A cross-sectional study with older people in Brazil found that social network (fewer meetings with friends and not having children) and social support (dissatisfaction/indifference with personal relationships) were independently associated with worse performance in ADL, but not the marital status [21]. Seeman et al. [40] found no significant protective effects for structural characteristics, including marital status, for disability in ADL among men. Wilcox et al. [41] found no significant differences in physical functionality between widowed and married women, as well as Unger et al. [42] found that being married did not predict changes in the functional capacity of men and women. Both studies were conducted in the United States of America (USA) and were longitudinal. When studying data from the USA census (cross-sectional design), Richmond & Roehner [43] found that compared to married people, widowed or single people had a higher prevalence of work disability. No systematic reviews or meta-analyses on the effect of marital status on functionality or studies in the Chilean population were found, but a study analyzing data from Latin America, including Chile, has found that living alone is a risk factor for functional limitation in data from cross-sectional studies, but not in data from longitudinal studies. This was the only aspect of social relations evaluated [19].

Both clusters identified as being at risk for functional limitation differ in the structural characteristics of the weaker social relations in relation to the reference cluster. These aspects provide opportunities for social influence, social engagement, person-to-person contact, and access to resources and material goods, which can impact health through behavioral, psychological, and physiological pathways [44]. Robles et al. [45] cites three possible explanations why marital status may impact health, which are: selection (people with better health and protective factors associated with better health may be more likely to marry/stay married), the resources provided by marriage (marriage allows access to joint economic, psychosocial and social benefits, which are not available to single people) and the stress associated with marital breakdown (divorce, separation or widowhood are stressful events). In addition, having a partner can protect health by providing greater social support and better health-related behaviors [46], apart from the fact that it can favor social integration [47]. Our analyses were adjusted for educational level and household income, so this possible mechanism would be considered. On the other hand, the fact that people without a partner participated very little in associations, clubs or organizations and also had less adherence to recreational activities confirms that, at least for our sample, having a partner can favor social integration.

The functional capacity of the population and its patterns vary between countries and within countries over time, without obvious explanation. In high-income countries there is some evidence of a reduction in total lifetime disability days, but in low- to middle-income countries there is no reliable evidence of a decline and even unhealthy life expectancy could be increasing. Uncertainty about the health of future generations persists in all countries, taking into account the different exposures to risk factors in different cohorts and the increases in the prevalence of chronic diseases [48].

Our study sought to approach aspects of the structure and function of social relations and form clusters of older people according to these aspects. Considering clusters formed is not free of limitations, since it does not allow us to study the aspects separately and depends on the methodology and reliability of the variables used to form the clusters. In our case, four of the five variables considered were dichotomous, which does not allow a more in-depth evaluation of the variability of these aspects and whether there are different relationships depending on level. Furthermore, the variables used do not include other relevant aspects such as the quality of relationships, social network size, frequency of encounters, reciprocity and conflicts in relationships. This study is also made up of older people from the urban area of ​​the Chilean capital, excluding rural older people, who represent 14.8% of the total number of people aged 60 or over in the country [49]. Another limitation is that we did not include the transition to death in our analyses. Strengths of the current study include, the longitudinal design, large sample size, different types of analysis adjusted for sociodemographic and health factors (such as depression). To our knowledge, this is the first study in the Chilean older adult population to analyze whether social relationships impact functionality in old age, through an analysis that seeks to integrate various aspects of social relationships.

## Conclusions

Our study showed that participating in social organizations, not living alone and living with a partner are protective factors for presenting and developing functional limitation in old age for community-living Chileans in an urban area. We know that recovery from disability can occur, so a better understanding of the disability process can direct strategies, programs or policies to groups of greater vulnerability in old age [50]. Older adults who are less socially integrated may be more vulnerable and face barriers when trying to access the benefits that social connections can offer, including health-related ones. To address this, we emphasize the need for studies that evaluate the impact of social relationships on the functionality of older adults in Latin American countries.

### Supplementary Information

Supplementary Material 1.

## Data Availability

The datasets produced and/or examined in the present study can be obtained from the corresponding author upon a reasonable inquiry.

## Abbreviations

ADL: Basic activities of daily living
IADL: Instrumental activities of daily living
AADL: Advanced activities of daily living
HR: Hazard ratio
CI: Confidence interval
SD: Standard deviation
USA: United States of America
FL: Funtional limitation
