# Supplementary figures and images for: Social relationships and their association with the functional capacity of older Chilean adults: longitudinal evidence
Source: BMC Geriatr. 2024 Jul 19;24:616. doi: 10.1186/s12877-024-05184-x (PMC11264702; doi:10.1186/s12877-024-05184-x)

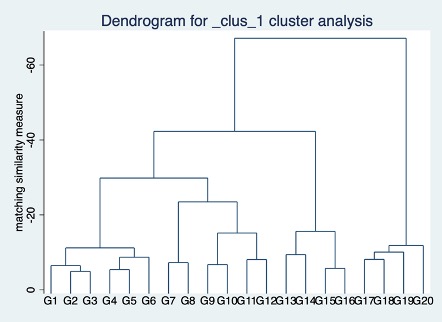

Supplement: Supplementary file 1 — Supplementary Material 1. [file 12877_2024_5184_MOESM1_ESM.jpg]
